# Internet-Based Self-Assessment for Symptoms of Internet Use Disorder—Impact of Gender, Social Aspects, and Symptom Severity: German Cross-sectional Study

**DOI:** 10.2196/40121

**Published:** 2023-01-12

**Authors:** Jan Dieris-Hirche, Laura Bottel, Stephan Herpertz, Nina Timmesfeld, Bert Theodor te Wildt, Klaus Wölfling, Peter Henningsen, Anja Neumann, Rainer Beckers, Magdalena Pape

**Affiliations:** 1 Department of Psychosomatic Medicine and Psychotherapy LWL-University Hospital, Ruhr University Bochum Bochum Germany; 2 Department of Medical Informatics, Biometry and Epidemiology Ruhr University Bochum Bochum Germany; 3 Psychosomatic Hospital Diessen Monastery Diessen Germany; 4 Outpatient Clinic for Behavioral Addictions Department of Psychosomatic Medicine and Psychotherapy University Medical Center of the Johannes Gutenberg-University Mainz Mainz Germany; 5 Department of Psychosomatic Medicine and Psychotherapy University Hospital Rechts der Isar Technical University Munich Munich Germany; 6 Institute for Health Care Management and Research University Duisburg-Essen Essen Germany; 7 Competence Centre of Healthcare Telematics Hagen Germany

**Keywords:** internet use disorder, eHealth, telemedicine, internet addiction, gaming disorder, OMPRIS, internet use, online self-assessment, self-assessment, eHealth services

## Abstract

**Background:**

Internet use disorder (IUD) is a new type of behavioral addiction in the digital age. At the same time, internet applications and eHealth can also provide useful support in medical treatment.

**Objective:**

The purpose of this study is to examine if an internet-based eHealth service can reach individuals with IUD. In particular, it should be investigated whether both male and female individuals with more severe IUDs can be reached.

**Methods:**

Data were retrieved from the OMPRIS (online-based motivational intervention to reduce problematic internet use and promote treatment motivation in internet gaming disorder and internet use disorder) project (DRKS00019925), an internet-based motivational intervention to reduce problematic internet use and promote treatment motivation in internet gaming disorder and IUD. During the recruitment process (August 2020-March 2022), a total of 3007 individuals filled out the standardized scale for the assessment of internet and computer game addiction (AICA-S). The assessment was accessible via the project homepage. There was no preselection of participants at this stage of the study; however, the offer was addressed to people with hazardous internet use and IUDs. The web-based assessment was free and could be found via search engines, but attention was also drawn to the service via newspaper articles, radio reports, and podcasts.

**Results:**

Out of 3007 who participated in the web-based self-assessment, 1033 (34.4%) are female, 1740 (57.9%) are male, 67 (2.2%) are diverse individuals, and 167 (5.5%) did not disclose their gender. The IUD symptom severity score showed a wide range between the AICA-S extreme values of 0 and 27 points. On average, the total sample (mean 8.19, *SD* 5.47) was in the range of hazardous IUD behavior (AICA-S cutoff>7.0). Furthermore, 561 individuals (18.7% of the total sample; mean 17.42, *SD* 3.38) presented severe IUD (AICA-S cutoff>13.5). Focusing on female and male participants, 20.9% (363/1740) of the men and 14.9% (151/1033) of the women scored above 13.5 points, which can be considered pathological IUD behavior (*χ*^2^_2,2773_=16.73, *P*<.001, effect size: Cramér *V*=0.078). Unemployment, being in vocational training or studying at a university, and being male were significantly associated with high IUD symptoms.

**Conclusions:**

Using a large sample, the study showed that both mildly and severely IUD-affected individuals can be reached via the internet. An internet-based eHealth offer can thus be a good way to reach patients with IUD where they are addicted—on the internet. In addition, eHealth services increase the likelihood of reaching female patients, who hardly ever come to specialized outpatient clinics and hospitals. Since social problems, especially unemployment, have a strong association with disease severity, the integration of social counseling into treatment seems advisable in terms of a multidisciplinary approach.

**Trial Registration:**

German Clinical Trials Register (DRKS) DRKS00019925; https://drks.de/search/de/trial/DRKS00019925

## Introduction

Internet use disorder (IUD) is a collective term defined as an uncontrolled and excessive use of different internet applications in terms of a behavioral addiction. Excessive internet use parallels other addictions. Several neural pathways in human additive behaviors, including IUD, are discussed, although the mechanisms are not yet clear [1]. IUD includes both excessive web-based gaming as the first officially acknowledged subtype and nongaming internet activities, that is, web-based shopping, pornography use, social network use, and generalized internet use [2]. The prevalence of IUD has increased in the last decades with a worldwide prevalence between 6% and 7%, with lower rates in northern and western Europe and higher rates in the Middle East [3,4]. In German populations, the IUD prevalence rates range between 1.2% and 3.1% [5-9]. Recent studies implicate that due to the COVID-19 pandemic, both the time spent on the internet and the prevalence of IUDs increased [10-14].

The effectiveness of eHealth interventions for patients with behavioral addictions is poorly studied. A systematic review from 2016 found a total of 16 studies testing internet-related interventions in substance addiction (11 studies in smoking, drinking, and opioid abuse) and behavioral addiction (5 studies in pathological gambling). Although only 5 of the 16 studies mentioned effect sizes (*d*=0.83-1.72), all studies reported positive treatment outcomes for their respective addictive behaviors [15]. So far, only few studies have investigated internet-based interventions for subjects with IUD and (internet) gaming disorder [16]. However, the first studies from health services research show significant therapeutic effects in symptom reduction and increasing the motivation to change [17]. Currently, study protocols are also published from the first ongoing randomized controlled trials (RCTs) to measure the effects of web-based therapy for IUDs [18,19]. However, it is still unclear if individuals having IUDs can be reached via the internet.

An RCT (Short-Term Treatment of Internet- and Computer Game Addiction [STICA]) on analogue psychotherapy in clinical patients with IUDs published in 2019 provided a good insight into the clinical history and internet usage patterns of IUD [20]. The authors examined a total of 143 patients diagnosed with IUD (intervention group n=72). The IUD diagnoses were standardized based on structured clinical interviews conducted by psychological and psychiatric experts. On average, the IUD patients scored above the cut-off score (13 points) of the Assessment of Internet and Computer Game Addiction Scale (AICA-S) and spent a mean time of 6.2 (SD 3.1) hours per day on the internet on weekdays and 8.0 (SD 3.8) hours per day on weekends [20]. However, this study was only carried out with men, as women rarely seek medical treatment in specialized outpatient clinics [21-23]. A German study from a specialized internet addiction outpatient clinic showed a share of women of less than 5% [24]. This gender gap appears to be of clinical relevance, as population-based studies have described that women and men are affected with approximately equal prevalence and severity [7] or only slightly increased prevalence in men [9,25]. Yet, a study of 327 female patients and 174 male patients from several German university psychosomatic outpatient clinics showed that 94.7% of IUDs were overlooked in female patients (compared to 66.6% in male patients) [26].

A review published in 2022 by leading experts in the research field still identifies major knowledge gaps that need to be closed. In particular, research is needed to understand the course and development of IUDs in different age groups and genders. In addition, the need for reliable methods for the early detection of people at risk, as well as preventive and therapeutic interventions, is stated [27,28].

This leads to the following research questions: (1) is it possible to reach individuals experiencing IUD symptoms via an internet-based health care service? (2) If this is possible, is the symptom exposure in a range that can be described as pathologic or clinically relevant? (3) Is there a gender difference in terms of accessibility? In particular, is it possible to reach female individuals with IUD symptoms as well? (4) If so, do female individuals differ from male individuals in terms of IUD severity, IUD prevalence, type of problematic internet application, and sociodemographic data?

## Methods

### Study Design and Procedure

Data was retrieved from the OMPRIS **(**online-based motivational intervention to reduce problematic internet use and promote treatment motivation in internet gaming disorder and internet use disorder) project (clinical trial registration no. DRKS00019925; ethics vote no. 19-6679), an internet-based motivational intervention to reduce problematic internet use and promote treatment motivation in internet gaming disorder and IUD [19]. The cross-sectional web-based assessment used in this analysis was the first stage of contact in the newly developed internet-based health intervention, which is currently being evaluated as an RCT. Anyone could take part in the assessment; there was no preselection of participants at this stage of the study. During the recruitment process (August 28, 2020, to March 11, 2022), a total of 3007 individuals took the AICA-S and provided media anamnesis and sociodemographic data via the study homepage. The assessment was free of charge and accessible via the OMPRIS project home page. The service could be found via search engines. In addition, newspaper articles, radio reports, and podcasts were used to draw attention to the offer. Media reports and podcasts offered free IUD screening and, if interested and indicated, participation in the OMPRIS intervention. After completing the screening test, which is the content of this study, the participants were offered both a detailed diagnosis using a standardized clinical interview and participation in an internet-based intervention. The results of the intervention study will be published elsewhere.

### Ethics Approval

The trial was registered in the German Clinical Trials Register (DRKS00019925) and was approved by the ethics committee for the Faculty of Medicine, Ruhr University Bochum, (approval 19-6779). All participants had to provide informed consent upon registration for the study.

### Assessment Instruments

#### The AICA-S Scale

The AICA-S scale [29,30] was used to assess the severity of the IUD. The scale consists of 14 items (on a 5-point Likert scale) that are related to the *Diagnostic and Statistical Manual of Mental Disorders*, Fifth Edition, criteria of substance use disorders and gambling disorder, and includes craving, loss of control, tolerance, unsuccessful attempts to spend less time on the internet, and withdrawal [31]. Furthermore, it assesses negative consequences in school, work, health, and with social partners. Moreover, time spent on the internet, the preferred web-based activities, and the preferred type of problematic internet use are requested. The cut-off is defined by statistical means based on epidemiological surveys and analyses [32]. A score of 7.0 to 13.0 points is rated as hazardous use or moderately addictive internet use. A score of 13.5 points or more is considered as pathological addictive internet use [33]. In addition to the items that make up the score, the AICA-S asks about the ranges of excessive internet use, with multiple answers possible. The following question was added in order to specifically capture the most problematic type of internet use: “Please select one: In your opinion, which of the web-based services mentioned is the most problematic to use?” Reliability of AICA-S (internal consistency of α=.89) and validity are determined in clinical and epidemiological surveys [20,34,35]. The AICA-S was successfully used in a recently published German RCT on the effectiveness of outpatient group therapy for IUD [20]. This study also showed good sensitivity to change after therapeutic intervention using self-assessment and assessment by experts [20].

#### Media Use and Sociodemographic Data

The average number of hours spent on the internet on weekdays and weekends was asked. In addition, subjective evaluations regarding problematic internet consumption was assessed. Furthermore, the type of internet use that is subjectively experienced as most problematic was asked. Individuals were also asked how they became aware of the web-based self-assessment. Finally, the following sociodemographic data were collected: age, gender, and current occupational situation.

### Statistical Analysis

Analyses were conducted with *SPSS Statistics for Macintosh* (IBM Corp). Descriptive data were reported using numbers, percentages, means, and SDs. We used the Kolmogorov-Smirnov test to test for normal distribution. However, due to the large number of participants, we decided to use parametric tests because they are very robust with a high *N* and show greater test power than nonparametric tests [36-38]. Independent *t* tests (2-sided) were used to compare gender differences (female vs male) for metric variables (effect size Cohen *d*). Chi-square tests with post hoc (adjusted residuals) tests were used for dichotomous and nominal variables (effect size φ for 2×2 tables and Cramér *V* for tables larger than 2×2). The Pearson product-moment correlation coefficient *r* was calculated for the association between 2 metric variables and the η coefficient for the association between metric (AICA-S score) and nominal (gender) variables. A 1-way *ANOVA* with Bonferroni correction was calculated to compare differences in more than 2 groups for metric variables (effect size η^2^). Multiple linear regressions (stepwise) were performed to assess the influence of sociodemographic variables on the AICA-S score. Furthermore, 2-way ANOVAs were performed to examine the impact and interaction effects of gender and sociodemographic variables on IUD symptom severity (AICA-S score). Effect sizes were defined following Cohen’s guideline [39]. Since there is officially a third gender, “Divers” in Germany, this was also asked and presented in the study, even though no a priori assumptions were made for this category. Thus, the focus of the reported statistical analyses was on the comparison between men and women.

## Results

### Demographics

The survey sample size (N=3007) varied over the period of 80 weeks between 5 (week 13) and 317 participations per week (week 26), with a mean of 37.59 (SD 42.91) participants per week. Demographics are shown in Table 1.

**Table 1:** Demographic data of the total sample (N=3007).

Variables		Values
**Gender, n (%)**		
	Female	1033 (34.4)
	Male	1740 (57.9)
	Divers	67 (2.2)
	Missing data^a^	167 (5.6)
**Age (years), n (%)**		
	<18	704 (23.4)
	18-24	744 (24.7)
	25-34	643 (21.4)
	35-44	359 (11.9)
	45-54	289 (9.6)
	55-64	163 (5.4)
	65-74	49 (1.6)
	>75	11 (0.4)
	Missing data^a^	45 (1.5)
Age (years), mean (SD)		29.17 (14.55)
**Occupational situation, n (%)**		
	Full-time employed	779 (25.9)
	Working part-time	274 (9.1)
	Self-employment	142 (4.7)
	Unemployed	285 (9.5)
	In vocational training	252 (8.4)
	Studying at the university	621 (20.7)
	Others (eg, pupil, retired, unable to work)	631 (21.0)
	Missing data^a^	23 (0.8)
**How did you become aware of the** **web-based** **offer?, n (%)**		
	Family and friends	433 (14.4)
	Internet search	864 (28.7)
	Medical institution	219 (7.3)
	Municipal institution	44 (1.5)
	School or university	521 (17.3)
	Press (radio, newspaper, etc)	521 (17.3)
	Others	361 (12.0)
	Missing data^a^	5 (0.2)

^a^Technical difficulty.

The results show that all 3007 individuals (1740 males, 1033 females, 67 divers individuals, and 167 participants who did not disclose their gender) could be reached during the 80-week web-based assessment, with 34.4% (n=1033) of the participants being women. About one-fourth of the participants were younger than 18 years (n=704, 23.4%), approximately another quarter ranged between 18 and 24 years old (n=744, 24.7%), and about one-fifth were aged between 25 and 34 years (n=643, 21.4%). The remaining participants were older than 35 years (n=871, 29.0%), with the higher age categories less frequently represented. Overall, 35.0% (n=1053) of participants were currently employed (full-time: n=779, 25.9%, part-time: n=274, 9.1%, students: n=621, and unemployed: n=285, 9.5%). A large proportion (n=863, 28.7%) of participants found the survey via internet search. However, referrals through family and friends (n=433, 14.4%), school or university (n=561, 18.7%), and press reports (n=521, 17.3%) were also relevant ways of contact.

### IUD Symptom Severity and Gender Differences

The general behavior regarding the exact type of internet use and web-based games, as well as the time spent on the internet, were asked. Furthermore, participants were asked whether they subjectively believed that they used the internet in a problematic way. Finally, symptoms of IUD were assessed. Table 2 shows the detailed results of internet use by male and female gender.

**Figure 1:**
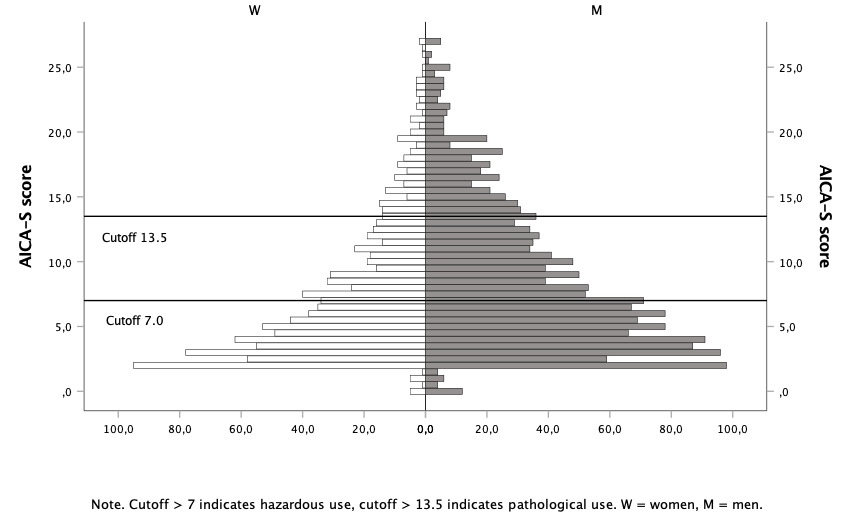
Frequency of AICA-S scores among women and men. AICA-S: Assessment of Internet and Computer Game Addiction Scale.

IUD symptom severity showed a wide range between the AICA-S extreme values of 0 and 27 points. Referring to the AICA-S cutoff, the total sample scored in the range of hazardous IUD behavior (mean 8.19, *SD* 5.47). Overall, 561 individuals (561/3007, 18.7%) could be classified into the group of pathological internet users and 927 individuals (927/3007, 30.8%) into the group of hazardous internet users. Male participants presented a slightly higher mean AICA-S score (mean 8.62, *SD* 5.57) than female participants (mean 7.45, *SD* 5.20). The difference between men and women, however, showed a small significant effect size (t_2771_=5.54, *P*<.001, *d*=0.215) and was of little clinical relevance (mean difference of 1.17 points in the AICA-S total score between men and women; both in the same clinical category according to the AICA-S cut-offs). Overall, 20.9% (363/1740) of the male and 14.6% (151/1033) of the female participants presented AICA-S scores of >13.5 points, which can be considered pathological IUD behavior. The differences in distribution were significant with small effect sizes (*χ*^2^_2,2773_=16.73, *P*<.001, Cramér *V*=0.078). Chi-square post hoc tests showed that male pathological users were significantly more frequent than expected according to the chi-square distribution (*P*<.001), while female pathological users were significantly less frequent than expected (*P*<.001). Figure 1 reports the frequencies of AICA-S scores of female and male participants.

Furthermore, differences in the AICA-S scores were investigated between the different types of problematic IUD. The level of IUD symptoms (as measured by the AICA-S total score) differed significantly for the different types of problematic IUD (Welch *F*_8,397.89_=23.12, *P*<.001). Table 3 shows the mean values and SDs of the AICA-S score for the different types of IUD.

On comparing the 3 AICA-S groups, inconspicuous use (mean 3.96, SD 1.52), hazardous use (mean 9.52, SD 1.87), and pathological use (mean 17.42, SD 3.38) by age, there were significant differences with higher age in the inconspicuous group (mean 30.8 years vs 27.6 years vs 27.8 years, respectively; Welch *F*_2,1470.87_=14.49, *P*<.001). Furthermore, significant differences were found among the 3 AICA-S groups with respect to current occupational situation (*χ*^2^_12,2984_=151.18, *P*<.001, Cramér *V*=0.121). It was found that pathological internet users (97/556, 17.5%) were significantly less likely to be full-time employed than hazardous internet users (231/921, 25.1%) and inconspicuous internet users (451/1507, 29.9%) but more likely to be unemployed (99/556, 17.8% vs 84/921, 9.1% vs 102/1507, 6.8%, respectively) or to be students (161/556, 29.0% vs 214/921, 23.3% vs 246/1507, 16.3%, respectively).

**Table 2:** Internet use and symptoms of IUD sorted by gender^a^.

Variables		Female participants (n=1033)	Male participants (n=1740)	*P* value		Effect size	
Age (years), mean (SD)		30.97 (14.56)	28.31 (14.30)	<.001		*d*=0.185	
Time (in hours) spent on the internet on a weekday (Mon-Fri), mean (SD)		4.92 (3.41)	5.91 (3.93)	<.001		*d*=0.266	
Time (in hours) spent on the internet on a weekend or holiday, mean (SD)		5.24 (3.26)	6.70 (3.92)	<.001		*d*=0.395	
Average calculated hours spent on the internet per week, mean (SD)		35.07 (21.64)	42.96 (25.23)	<.001		*d*=0.252	
**How often are you using the internet?, n (%)**					.21		Cramér *V*=0.046
	Every day	1005 (97.3)	1689 (97.1)				
	2-3 times per week	19 (1.8)	41 (2.4)				
	1 time per week	7 (0.7)	3 (0.2)				
	1 time per month	1 (0.1)	3 (0.2)				
	Less than 1 time per month	1 (0.1)	3 (0.2)				
**For how long (in hours) do you usually use the internet?, n (%)**					<.001		Cramér *V*=0.165
	<1	155 (15.0)	136 (7.8)				
	1-2	279 (27.0)	362 (20.8)				
	3-4	267 (25.8)	473 (27.2)				
	5-6	184 (17.8)	347 (19.9)				
	>6	148 (14.3)	422 (24.3)				
AICA-S^b^ total sample, mean (SD)		7.45 (5.20)	8.62 (5.57)	<.001		*d*=0.215	
**AICA-S severity groups, n (%)**					<.001		Cramér *V*=0.098
	Unproblematic use	579 (56.1)	815 (46.8)				
	Hazardous use	303 (29.3)	562 (32.3)				
	Pathological use	151 (14.6)	363 (20.9)				
**In your opinion, which web-based offers do you use excessively? (Multiple answers are possible), n (%)**							
	Web-based games (eg, role-playing games and ego shooters)	169 (16.4)	838 (48.2)	.048		*φ*=0.061	
	Web-based shopping (eg, eBay and Amazon)	187 (18.1)	184 (10.6)	.22		φ=0.059	
	Chats or forums	267 (25.9)	329 (18.9)	.34		φ=0.038	
	Writing emails	76 (7.4)	99 (5.7)	.40		φ=0.057	
	Web-based sex (eg, pornographic material)	16 (1.6)	393 (22.6)	<.001		φ=0.297	
	Web-based gambling (eg, poker, casinos, betting)	15 (1.5)	52 (3.0)	.40		φ=0.087	
	Web-based communities (eg, Facebook and Instagram)	437 (42.3)	435 (25.0)	.10		φ=0.055	
	Information searching (eg, Wikipedia)	190 (18.4)	294 (16.0)	.25		φ=0.049	
	Web-based streaming services (eg, Netflix, Amazon Prime, YouTube)	577 (55.9)	963 (55.3)	>.99		φ=0.000	
	Others	67 (6.5)	118 (6.8)	.02		φ=0.152	
**In your opinion, the use of which of the web-based services mentioned is the most problematic?, n (%)**					<.001		Cramér *V*=0.369
	Web-based games (eg, role-playing games and ego shooters)	124 (12.0)	515 (29.6)				
	Web-based shopping (eg, eBay and Amazon)	73 (7.1)	49 (2.8)				
	Chats or forums	66 (6.4)	42 (2.4)				
	Writing emails	12 (1.2)	14 (0.8)				
	Web-based sex (eg, pornographic material)	29 (2.8)	276 (15.9)				
	Web-based gambling (eg, poker, casinos, and betting)	89 (8.6)	174 (10.0)				
	Web-based communities (eg, Facebook and Instagram)	279 (27.0)	167 (9.6)				
	Information searching (eg, Wikipedia)	68 (6.6)	66 (3.8)				
	Web-based streaming services (eg, Netflix and YouTube)	293 (28.4)	437 (25.1)				

^a^Missing values for gender: n=167. We excluded participants with the “divers” gender (n=67).

^b^AICA-S: Assessment of Internet and Computer Game Addiction Scale.

**Table 3:** Assessment of Internet and Computer Game Addiction Scale (AICA-S) scores differentiated by type of internet use disorder^a^.

Variable	Participants, n	AICA-S score, mean (SD)
Web-based streaming	795	9.01 (5.57)
Web-based gaming	678	9.23 (5.82)
Social networks or communities	493	7.76 (4.97)
Web-based pornography or sex	333	8.22 (5.43)
Web-based gambling	283	5.77 (3.68)
Information search	145	7.48 (5.32)
Web-based shopping	128	5.98 (4.89)
Web-based chats	121	8.40 (6.37)
Emails	27	6.44 (5.08)

^a^Missing values: *n*=4.

### Gender Differences in Time Spent on the Internet and Types of Problematic Internet Use

Male participants used the internet for 5.91 (*SD* 3.93) hours per day on weekdays and 6.70 (*SD* 3.92) hours per day on weekends (calculated weekly time of use 42.96 hours, *SD* 25.23 hours). Female participants used the internet 4.92 (*SD* 3.41) hours per day on weekdays and 5.24 (*SD* 3.26) hours per day on weekends (calculated weekly time of use 35.07 hours, *SD* 21.64 hours). The differences in usage time reached significance, (weekdays: t_2771_=6.76, *P*<.001; weekends: t_2771_=10.06, *P*<.001) with small effect sizes (*d*=0.252-0.395).

The most frequently mentioned types of excessive internet use among female users were (multiple answers possible) web-based streaming (577/1033, 55.3%), social networking (437/1033, 42.3%), and chats or forums (267/1033, 25.9%). In contrast, the most frequently mentioned types of excessive internet use for men were web-based streaming (963/1740, 55.3%), gaming (838/1740, 48.2%), and web-based pornography (393/1740, 22.6%). The largest significant gender difference with moderate effect size was found for web-based pornography (*χ*^2^_1, 451_=39.77, *P*<.001, φ=0.297; for further details, see Table 2).

### Correlations

Table 4 shows the correlations between the IUD symptoms (AICA-S score), usage time (h/wk) and age within the total sample of 3007 participants.

**Table 4:** Descriptive statistics and correlation for internet use disorder symptoms.

Variable	Participants, n	AICA-S^a^ score, mean (SD)	Correlations, *r*	
			AICA-S score	Time of use per week (h)
AICA-S score	3007	8.20 (5.47)	—^b^	—
Time of use per week (h)	3007	39.95 (24.33)	0.523^c^	—
Age	2962	29.17 (14.55)	–0.090^c^	–0.164^c^

^a^AICA-S: Assessment of Internet and Computer Game Addiction Scale.

^b^Not determined.

^c^Correlation is significant when *P*=.01 (2-tailed).

The association between gender and IUD symptoms (AICA-S) was significant (η=0.098, η^2^=0.0096, *P*<.001), meaning that only 0.96% of the variance regarding the AICA-S could be explained by gender (small effect). Furthermore, the association between gender and time spent on the internet (h/wk) was significant (η=0.157, η²=0.0246, *P*<.001), meaning that only 2.46% of the variance in usage time could be explained by gender (small effect).

### Effect of Sociodemographic Factors and Gender on the AICA-S Score

Stepwise multiple regression analysis was performed within the total sample to test if sociodemographic factors including age, occupational status (divided into 3 categories: employed or self-employed, unemployed, and being in vocational training or studying) and gender (male or female) were significantly associated with IUD symptom severity. The following characteristics were chosen as references for the dummy variables: female gender and occupational status (employed or self-employed).

A significant regression equation was found (*F*_3,2317_=33.44, *P*<.001), with an *R^2^* of 0.042 for the final model. Furthermore, being unemployed (B=3.170, SE 0.38; *β*=.175; *P*<.001), being in vocational training or studying at a university (B=1.054, SE 0.25; *β*=.088; *P*<.001), and being male (B=1.046, SE 0.23; *β*=.092; *P*<.001) were significant factors in the final model, while age (*P*=.90) did not reach significance. A participant's predicted AICA-S score increased by 3.17 points if the participant was unemployed, by 1.05 points if the participant was in vocational training or studying, and by 1.05 points if the participant was a man.

**Figure 2:**
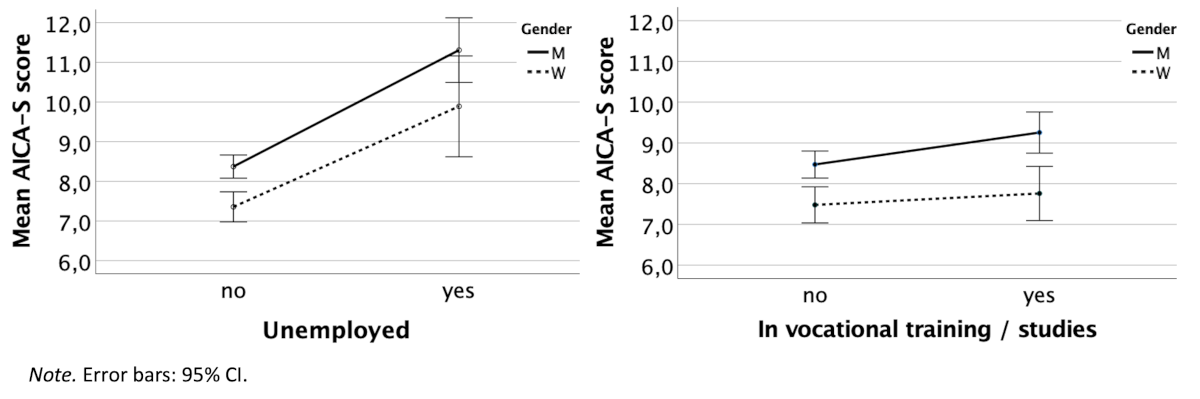
Two-way ANOVA: main and interaction effects of gender and unemployment (left) or being in vocational training or studying (right). AICA-S: Assessment of Internet and Computer Game Addiction Scale.

Due to the significant influence of unemployment and vocational status, two 2-way ANOVAs were conducted to explore the main and interaction effects of unemployment (yes or no), being in vocational training or studying (yes or no), and gender (female or male) on levels of IUD symptom severity (AICA-S score). First, there was a significant main effect for unemployment (*F*_1,2318_=45.78, *P*<.001, η²=0.019). Thus, unemployed participants had higher AICA-S scores. In addition, there was a significant main effect for gender (*F*_1,2318_=9.06, *P*=.003, η²=0.004), meaning that males had higher AICA-S scores. However, the interaction effect between unemployment and gender was not significant (*F*_1,2318_=0.25, *P*=.62). Second, there was a small significant main effect for being in vocational training or studying (*F*_1,2318_=4.35, *P*=.04, η²=0.002). Thus, participants who were in vocational training or studying had higher AICA-S scores. In addition, there was a significant main effect for gender (*F*_1,2318_=23.73, *P*<.001, η²=0.010), meaning that males had higher AICA-S scores. However, the interaction effect between being in vocational training or studying and gender was, again, not significant, (*F*_1,2318_=0.972, *P*=.32). Thus, in summary, there were no interaction effects between gender and the other sociodemographic variables, although all the main effects were significant (Figure 2).

## Discussion

### Principal Findings

First, the results showed that there was continuous participation in the web-based assessment during 80 weeks, with a very wide range in IUD symptom burden (between 0 and 27 points) reported (research question 1). The high number of 3007 participants showed that the web-based offer was generally accepted. The mean symptom burden of the total group was 8.19 (SD 5.47), indicating *hazardous* internet use. Compared to the sample of the STICA study [20], the average score in our sample was lower (using the same AICA-S questionnaire) but in the diagnostic category of hazardous internet use. This can be explained by the fact that in the STICA study, explicitly diagnosed patients were treated, whereas in our study, access was open to all affected individuals who suspected a problem in their internet use, which is a more at-risk group. However, this indicates that web-based assessment can be used as an eHealth offering for both prevention and treatment. Furthermore, the high prevalence of users with pathological internet use (561/3007, 18.7%) in our sample shows that web-based self-assessment also reaches patients with clinically relevant disease severity (research question 2). The proportion of users with pathological internet use was considerably higher than the prevalence figures in the general German population [7]. However, it should be discussed that our study was conducted at the time of the COVID-19 pandemic, and the prevalence of problematic internet use might have been subsequently increased [14]. The assessment was freely offered on the internet, and anyone interested could take part in the screening. This means that the participants were not selected in a representative way and were probably people who suspected a problem with their internet use. Therefore, we assume that our sample is not representative but rather represents a risk population. Nevertheless, our results show that a free web-based self-assessment reaches people having IUD with both severe and mild pathologies. Thus, this offer can actually be a low-threshold offer to reach affected patients with IUD who may still shy away from real contact with an outpatient clinic. This confirms the initial findings from our working group, which indicated a good response to an internet-based support [17]. Our study found a significant association between time spent on internet use and IUD symptomatology, which had also been demonstrated in a previous study by our research group on a similar large high-risk sample [40]. Age, however, was not significantly associated with higher symptom scores, which contradicts a previous representative study from Germany in which young internet users were significantly more often affected by IUD [6]. This could be explained by the influence of the COVID-19 pandemic, which also caused many older people to spend more time at home.

With regard to research question 3, it could be shown that both women and men used the service of a web-based self-assessment for IUD symptoms. The proportion of women participating (1033/3007, 34.4%) was much higher than expected and exceeded the proportion of women who visited IUD specialist outpatient clinics in Germany [24]. Female individuals with hazardous internet use are thus very reachable via the internet. Moreover, our results showed that within the high-risk study sample, women were less likely to exhibit pathological internet use than men (prevalence: 151/1033, 14.6% vs 363/1740, 20.9%). These findings support some recent systematic reviews examining the global prevalence of IUD and IGD, which found an increased pooled prevalence in men [9,25]. Other representative studies, however, found no gender differences in IUD prevalence [6].

Regression analysis showed that the male gender was significantly associated with the AICA-S score. The effect of gender, however, was less significant than the effect of unemployment. In terms of symptom severity, other sociodemographic variables appear to have had an even greater effect than the question of gender. Furthermore, there were no interaction effects of gender and sociodemographic factors on symptom severity. As a result, it mattered whether someone was a man or woman, or unemployed or working. However, the interaction effect was not significant. The results confirm previous findings describing unemployment as a risk factor in IUD and gaming disorders [7,41]. The fact that vocational training and study was associated with high IUD symptoms could also be related to the COVID-19 pandemic, since home office often replaced analog presence. The results suggest that it may be helpful to integrate social counseling into treatment planning for patients with IUDs to work out occupational options. Furthermore, gender differences were found regarding the most frequent problematic internet applications. In line with the existing research literature, women had higher affinity for social networks and social media, while men were more likely to play computer games [7,42,43]. Other studies discuss a more complex view that a combination of female gender, more disinhibited, neurotic, narcissistic, and extraverted patterns, and lower body satisfaction is associated with increased risk for internet addiction [44]. Surprisingly, however, many individuals (men and women) in our study stated web-based streaming as the main problem. As the widespread availability of web-based streaming services is a recent development, this is less often considered in the current research literature on behavioral addictions. On the other hand, there is a lot of recent research literature that examines binge-watching and changing media behavior, particularly during the COVID-19 pandemic [10,45,46].

A significant difference was found in the subjective problematic use of pornography. Significantly more men (393/1740, 22.6%) reported this; pornography was less often relevant for women (16/1033, 1.6%). This is in line with previous research literature and experience from therapeutic practice [47]. Gender-specific IUD treatment approaches may be relevant in the specific IUD of pathological pornography use.

In summary, and with regard to research question 4, there are some differences with small effect sizes between men and women with regard to IUD symptom severity (mean differences of 1.17 AICA-S points). At most, there are individual gender differences in the type of problematic internet use. A secondary finding was that being unemployed as well as undergoing vocational training or studying at a university were significantly associated with IUD. It can be assumed that the free time allocation promotes a higher usage time. Perhaps being at home (possibly alone because others are busy) or not working in a specific workspace or outside the home plays an important role. It is possible that being at work is a protective factor, as has been reported in a previous study [7]. However, we cannot exclude at this point that any other activity outside one’s home or with other people could lead to similar results.

### Limitations

The study was conducted on a large population using a questionnaire. The cutoff of the questionnaire was used to classify the severity. This type of diagnosis does not replace a clinical diagnosis by an expert and is therefore naturally prone to false positives or false negatives. However, numerous population-based studies have been conducted using this methodology. In selecting the questionnaire, an established instrument that is more clinically oriented and makes a conservative assessment was chosen. Moreover, it was used as the main outcome in a previous high-quality RCT [20], which allows for good comparability of clinical characteristics. Furthermore, we use a cross-sectional design, which can only make limited statements regarding the question of causality. Regression analyses in cross-sectional samples should in most cases be interpreted as associations, and casual attributions should be made with caution. In our study, we used an established diagnostic scale based on the *Diagnostic and Statistical Manual of Mental Disorders,* Fifth Edition, criteria for internet gaming disorder published in 2013 [30]. In 2022, however, a new questionnaire was developed, which for the first time is based on the new diagnostic criteria according to the *11th revision of the International Classification of Diseases* [48,49]. Future studies should therefore also include instruments that reflect the criteria of the *11th revision of the International Classification of Diseases*.

### Conclusions

Using a very large sample, the study shows that both people with mild and those with severe IUD can be reached via the internet. Thus, a free eHealth offer can be a good way to reach patients with IUD where they are addicted—on the internet. In addition, eHealth services increase the likelihood of reaching female patients, who, by analogy, hardly ever come to specialized outpatient clinics and hospitals. Nevertheless, men appeared to be affected by IUD symptoms somewhat more frequently and severely than women, although the differences tended to have small effect sizes. Since social problems, especially unemployment, have a strong effect on disease severity, the integration of social counseling into treatment seems advisable in terms of a multidisciplinary approach.

## Abbreviations

AICA-S: Assessment of Internet and Computer Game Addiction Scale
IUD: internet use disorder
OMPRIS: online-based motivational intervention to reduce problematic internet use and promote treatment motivation in internet gaming disorder and internet use disorder
RCT: randomized controlled trial
STICA: short-term treatment of internet- and computer game addiction
